# The Prevalence of Psychological Distress and Its Relationship to Sleep Quality in Saudi Arabia's General Population During the COVID-19 Pandemic

**DOI:** 10.3389/fpsyt.2021.809040

**Published:** 2022-02-03

**Authors:** Maha M. AlRasheed, Sinaa Al-Aqeel, Afnan M. Alkadir, Khulood Bin Shuqiran, Fowad Khurshid, Noura M. AlRasheed, Roua M. Al-kufeidy, Omar A. Alhaj, Haitham Jahrami, Ahmed S. BaHammam

**Affiliations:** ^1^Department of Clinical Pharmacy, College of Pharmacy, King Saud University, Riyadh, Saudi Arabia; ^2^Princess Nourah Bint Abdulrahman University, Riyadh, Saudi Arabia; ^3^General Administration of School Health, Ministry of Health, Riyadh, Saudi Arabia; ^4^Department of Botany and Microbiology, Molecular Immunology, College of Science, King Saud University, Riyadh, Saudi Arabia; ^5^Department of Nutrition, Faculty of Pharmacy and Medical Sciences, University of Petra, Amman, Jordan; ^6^Ministry of Health, Manama, Bahrain; ^7^College of Medicine and Medical Sciences, Arabian Gulf University, Manama, Bahrain; ^8^Department of Medicine, College of Medicine, University Sleep Disorders Centre, King Saud University, Riyadh, Saudi Arabia; ^9^The Strategic Technologies Program of the National Plan for Sciences and Technology and Innovation in the Kingdom of Saudi Arabia, Riyadh, Saudi Arabia

**Keywords:** PSQI score, K10 score, COVID-19, psychological distress, sleep quality

## Abstract

**Background:**

We aimed to examine the effect of the COVID-19 pandemic and associated mitigation measures on sleep quality and psychological distress in Saudi Arabia.

**Methods:**

Subjective sleep quality over the preceding 30 days was measured using the Pittsburgh Sleep Quality Index (PSQI). In addition, Kessler Psychological Distress Scale (K10) was used to assess the psychological distress.

**Results:**

The study included 836 participants. The median age was 28 years, 624 (74.64%) were females, and 158 (18.90%) were healthcare workers. Factors associated with poor sleep were recent changes in the sleep habits *p* = 0.004), anxiety or fear because of coronavirus news on social media *p* = 0.02), fear because there was no approved drug to treat COVID-19 *p* = 0.03), and unaware of the presence of chronic diseases *p* = 0.03). Female gender *p* = 0.02), fear or anxiety because of coronavirus news on social media *p* = 0.04), recent change in sleep habits (OR: 1.97 (1.15–3.39); *p* = 0.01), fear because there is no approved drug to treat COVID-19 *p* = 0.001), monthly income <1000 SR *p* = 0.01), and isolation *p* = 0.01) were associated with distress. PSQI and K10 scores were significantly correlated *p* < 0.001).

**Conclusion:**

Poor sleep and psychological distress are common during the COVID-19 outbreak in Saudi Arabia. Identifying factors associated with poor sleep and psychological distress would help develop specific intervention programs that enhance mental health and sleep quality during pandemics.

## Introduction

The World Health Organization declaration (1) on March 11, 2020, classifying the severe acute respiratory syndrome coronavirus 2 (SARS-CoV-2) disease as a global pandemic mandated governments worldwide to implement measures to mitigate virus spread. These procedures included lockdowns, quarantine, social distancing, and travel restrictions, all of which may reduce physical activity and exposure to daylight, adversely affecting the pace of time flow (2) and disrupting night-time sleep (3, 4). These measures increase the risk of mental health problems. A rise in the prevalence of generalized anxiety disorders, post-traumatic distress, depression, and worsening of psychiatric symptoms has been reported in published systematic reviews (5–9) because of the pandemic. In addition, female gender, younger age, unemployment, educational level, insufficient knowledge of the disease, frequency of exposure to social media/disease-related news, and chronic/psychiatric illnesses have been implicated as risk factors for these disorders. A meta-analysis of studies up to July 5, 2020, identified 44 publications involving 54,231 participants from 13 countries, demonstrating a pooled global rate of 35.7% for sleep problems among the studied populations. Patients infected with SRS-CoV-2, commonly known as COVID-19, exhibited a higher rate of sleep problems of 74.8% compared to 36.0% in healthcare workers and 32.3% in the general population (10). Examples of reported sleep problems linked to the COVID-19 pandemic involve increased sleep duration and latency (11), worsening of sleep quality (11–16), decrease in the amount and regularity of sleep, and insomnia symptoms (13).

Gender has been demonstrated to play a major effect in the experience of sleep disruptions in previous research (17). A study of research, for example, discovered that females have a greater risk of insomnia than males. Matud and Garca (18) found that women had a greater frequency of mental health concerns than males. Furthermore, sleep loss leads women to be more anxious than males (19). During the COVID-19 epidemic, females experienced more psychological anguish than males (20). However, whether gender influences the association between sleep problems and mental health during the COVID-19 pandemic is unknown and warrants more investigation. As a result, we expected that gender would have an influence on the connection between sleep disruptions and mental health.

Saudi Arabia has implemented several mitigation measures such as curfew, self-quarantine for infected or symptomatic individuals and travelers arriving in Saudi Arabia, mandatory face masks, and restrictions on national and international journeys since the identification of the first case in March 2020 (21). Full and partial curfews were imposed from March 24 until June 20, 2020. Lockdown included schools, universities, and shops not selling basic stuff. While the published research on mental health and sleep quality and its association with COVID-19 might apply to Saudi Arabia, many country-specific social and economic variables could influence the rates of mental and sleep problems. Hence, local decision-makers need local data to plan preventive public health interventions during potential subsequent pandemics. Additionally, there is a need to identify risk factors associated with mental and sleep problems to prioritize preventive and treatment strategies targeting vulnerable groups (22).

During the COVID-19 epidemic, the current study looked at the incidence of sleep disruptions in different demographic categories of Saudi Arabians. Furthermore, the association between sleep disruptions and mental health status, as well as the factors that influence it, was investigated. The current study's findings will give vital information to medical personnel and the government regarding who would benefit the most from initiatives aimed at minimizing sleep disruptions and promoting mental health during the COVID-19 crisis.

## Methodology

### Study Design and Participants

A cross-sectional questionnaire-based study was conducted between May 8 and June 29, 2020. The study was conducted during the lockdown period in Saudi Arabia. The eligible study population included participants aged 18 years and older, capable of reading and understanding the questionnaire that was availed to participants in Arabic and English to select their preferred language and those living in Saudi Arabia during the study period. We excluded participants on sleep or psychiatric medications (*n* = 76). A convenience sampling technique was employed to recruit the participant according to availability and accessibility. The required sample size for the study was calculated using the Raosoft sample size calculator, employing a type I error margin of 5% and a confidence interval of 95%, power was set at 80% (23) and the population of Saudi Arabia estimated at 34,813,871 according to the United Nations database (24). An estimated sample size of 385 individuals was determined as adequate for the study. However, to increase power, the ultimately recruited population was 836.

This study was approved by the Institutional Review Board (IRB) of King Saud University Medical City (E-20-4869) and the IRB of the Ministry of Health (20-331E).

### Survey Instruments

The principal investigator constructed a dedicated account for the online questionnaire using a Google Form. It collected information on (Supplementary Table 1):

a. Socio-demographic characteristics of the participants, including age, gender, marital status, work sector, family status, income, education, employment status, and region of residence.
b. The social interaction involved attitude and response to social events, measured with the desire to attend such events, attendance frequency, and involvement in the activities.
c. COVID-19 and associated disease data aimed to evaluate participants' personal experience with COVID-19 infection. The questions used to cover this item involved “the frequency of going out before the coronavirus pandemic, information about coronavirus and its ways of spreading, concerns about lack of approved drug to treat COVID-19, the effect of COVID-19-related news on social media on anxiety, and fear, whether infected by COVID-19, suffering from a chronic disease, pregnant or how one's sleeping habits were affected by special occasions such as the month of Ramadan or vacations.” A recent meta-analysis showed that Ramadan and related behaviors influence sleep duration and daytime drowsiness. The average total sleep time for the entire population was 7.2 h at the start of the study, which fell by around 1 h throughout Ramadan. Ramadan fasting might affect daytime drowsiness, although the effect is minimal, as reflected by a recent meta-analysis that showed nearly a 1 point increase in the ESS score (25).
d. Over the preceding 30 days, subjective sleep quality was measured using the Pittsburgh Sleep Quality Index (PSQI) (26). The tool looks at seven areas: subjective sleep quality, sleep latency, sleep duration, habitual sleep efficiency, sleep disturbances, the use of sleep-promoting medication, and daytime dysfunction (26). Each component is scored on a four-point scale from 0 (no difficulty) to 3 (severe difficulty). The global score is calculated by adding each component's score, ranging from 0 to 21, with higher scores indicating lower sleep quality. It presents a cut-off point of PSQI ≤ 5 as good and PSQI >5 as poor sleep quality (26). We used the Arabic version of the PSQI got from MAPI Research Trust. The validity and reliability of the Arabic version have been demonstrated (27). The PSQI has a sensitivity of 89.6% and specificity of 86.5% for distinguishing good and poor sleepers, using a cut-off score of 5.
e. Kessler Psychological Distress Scale (K10) measures psychological distress based on ten questions assessing emotional states (28). It uses a 5-point scale ranging from “None of the time,” which is assigned a score of 1, to “All of the time,” assigned a score of 5. The maximum total score is 50, while the minimum is 10. A total score of <20 was considered not to represent stress of any level, while 20–24 represented mild stress, 25–29 moderate stress, and 30–50 represented severe stress (28). We used the Arabic version of K10 obtained from the Health Translation online library (29). The validity and reliability of the Arabic version have been demonstrated (30).

### Procedure

The principal investigator posted an invitation on Twitter, WhatsApp, and Facejournal. We reached isolated participants by sending invitations to special governmental facilities to be shared with them. Participants responded to the survey by scanning the Quick Response code (Q.R. code) on the questionnaire address or clicking on the appropriate link. Before taking part in the study, the participants gave their informed consent. There were no monetary or non-monetary incentives for time or responses; participation was voluntary.

### Statistical Analysis

Data visualization and Shapiro-Wilk normality test was employed to evaluate the distribution of the continuous variables. Non-normally distributed continuous data were expressed as median (25th−75th percentiles) and compared using the Mann-Whitney U test. Categorical data were presented as numbers and percentages and compared by the Chi-square test or Fisher's exact test if the expected frequency was less than five.

The correlation between the K10 and PSQI scores was tested using the Spearman correlation test. Multivariable logistic regression analysis was used to identify risk factors associated with poor sleep and distress. Univariable logistic regression was performed for the individual variables, whereby those displaying a *P*-value <0.2 were included in a stepwise logistic regression analysis with a forward selection. A stay *P*-value of < 0.05 was required to be included in the final regression model. Collinearity was tested using variance inflation factor (VIF), model calibration with the Hosmer-Lemeshow test, and discrimination with the area under the receiver operator curve. Negative binomial regression was used to identify factors associated with PSQI and K10 scores. We followed the same route for model selection as described for logistic regression analysis. Odds ratios (OR) and incidence rate ratios (IRR) were reported for the logistic and negative binomial regression models, respectively. Marginal analysis was performed after negative binomial regression to identify the K10 scores predicting PSQI scores. A generalized structural equation modeling was used to test the relationship between poor sleep and distress in the presence of other variables that could affect sleep. All statistical analyses were performed using STATA 16.1 (Stata Corp- College Station- TX- USA). A *P*-value of ≤ 0.05 was considered statistically significant.

## Results

### Participants

One thousand three hundred fifty-four participants opened the survey; 913 completed it, and 441 did not. After excluding the participants on sleep or psychiatric medications, 836 were included. There were no differences in age (*p* = 0.67), gender (*p* = 0.63), marital status (*p* = 0.70), area of residence (*p* = 0.56), job-status (*p* = 0.47), education (*p* = 0.92) and nationality (*p* = 0.15) between respondents and non-respondents. However, non-respondents were more among healthcare professionals and had higher income (*p* < 0.001).

### Socio-Demographics

We included 836 participants in our analysis. The median age was 28 years (25th−75th percentiles: 22–38), and 624 (74.6%) were females. Healthcare workers represented 18.9% of our participants (*n* = 158) and the majority were Saudis nationals (*n* = 775, 92.7%) and live in Riyadh (*n* = 536, 64.1%) (Table 1).

**Table 1:** Socio-demographic of participants.

**Variable**	**All participants (*n* = 836)**
Age (Years)	28 (22–38)
Female	624 (74.64)%
**Marital status**	
Single	464 (55.5%)
Married	336 (40.19%)
Divorced/widow/separated	36 (4.31%)
Do you work in the healthcare sector? (Yes)	158 (18.9%)
Do you have children?	322 (38.52%)
**How many members of your family live with you at home (including you)?**	
One to two persons	103 (12.32%)
Three to five persons	270 (32.30%)
More than five persons	463 (55.38%)
**Nationality**	
Saudi	775 (92.7%)
Non-Saudi	61 (7.3%)
**Educational level**	
Middle school or lower, High school or Diploma	196 (23.44%)
Bachelor's degree or higher	640 (76.56%)
**Job-status**	
I do not work	159 (19.02%)
Employee	341 (40.79%)
Self-employed	22 (2.63%)
Student	314 (37.56%)
**Monthly income**	
I don't want to answer	315 (37.68%)
<1000 SR	136 (16.27%)
1000–2999 SR	81 (9.69%)
3000–5999 SR	44 (5.26%)
6000–9999 SR	57 (6.82%)
10000–30000 SR	171 (20.45%)
>30000	32 (3.83%)
Region of residence (Riyadh)	536 (64.11%)
**Social interaction**	
Loves and waits for social events	301 (36%)
Gets bored of social events and does not go there	162 (19.38%)
Hates social events and does not go there	54 (6.46%)
Neutral	319 (38.16%)
**How often do you go out weekly before the coronavirus pandemic outside working hours?**	
None	75 (8.97%)
Once a week	178 (21.29%)
Two to three times a week	325 (38.88%)
Four times or more	258 (30.86%)
**I have good information about coronavirus and its ways of spreading**	
Highly agree	733 (87.68%)
Agree	20 (2.39%)
Neutral	68 (8.13%)
Disagree	12 (1.44%)
Highly disagree	3 (0.36%)
**I feel very afraid because there is no approved drug to treat COVID-19**	
Highly agree	152 (18.18%)
Agree	296 (35.41%)
Neutral	200 (23.92%)
Disagree	148 (17.7%)
Highly disagree	40 (4.78%)
**Coronavirus news on social media increases my anxiety and fear**	
Highly agree	169 (20.22%)
Agree	271 (32.42%)
Neutral	176 (21.05%)
Disagree	173 (20.69%)
Highly disagree	47 (5.62%)
Isolated	84 (10.05%)
**Do you have COVID-19?**	
Yes	24 (2.87%)
No	745 (89.11%)
In the past	67 (8.01%)
**Curfew hours during the past month**	
Partial curfew 6 a.m.−3 p.m.	424 (50.72%)
Partial curfew 6 a.m.−8 p.m. Penalties for not wearing a face mask	409 (48.92%)
No curfew, Penalties for not wearing a face mask, refuse to be checked for temperature	3 (0.36%)
Pregnancy	19 (2.27%)
Are your sleep habits affected by special occasions as Ramadan or vacations? (Yes)	768 (91.87%)
**Do you suffer from a chronic disease?**	
No	671 (80.26%)
Yes	105 (12.56%)
I don't know	60 (7.18%)

*Continuous data were expressed as median (25th−75th percentiles) and categorical data as numbers and percentages*.

The socio-demographic data and the questionnaire responses were compared between participants with poor vs. good sleep and participants who had distress vs. those without distress in Supplementary Table 1.

### PSQI and K10 Scores

**Figure 1:**
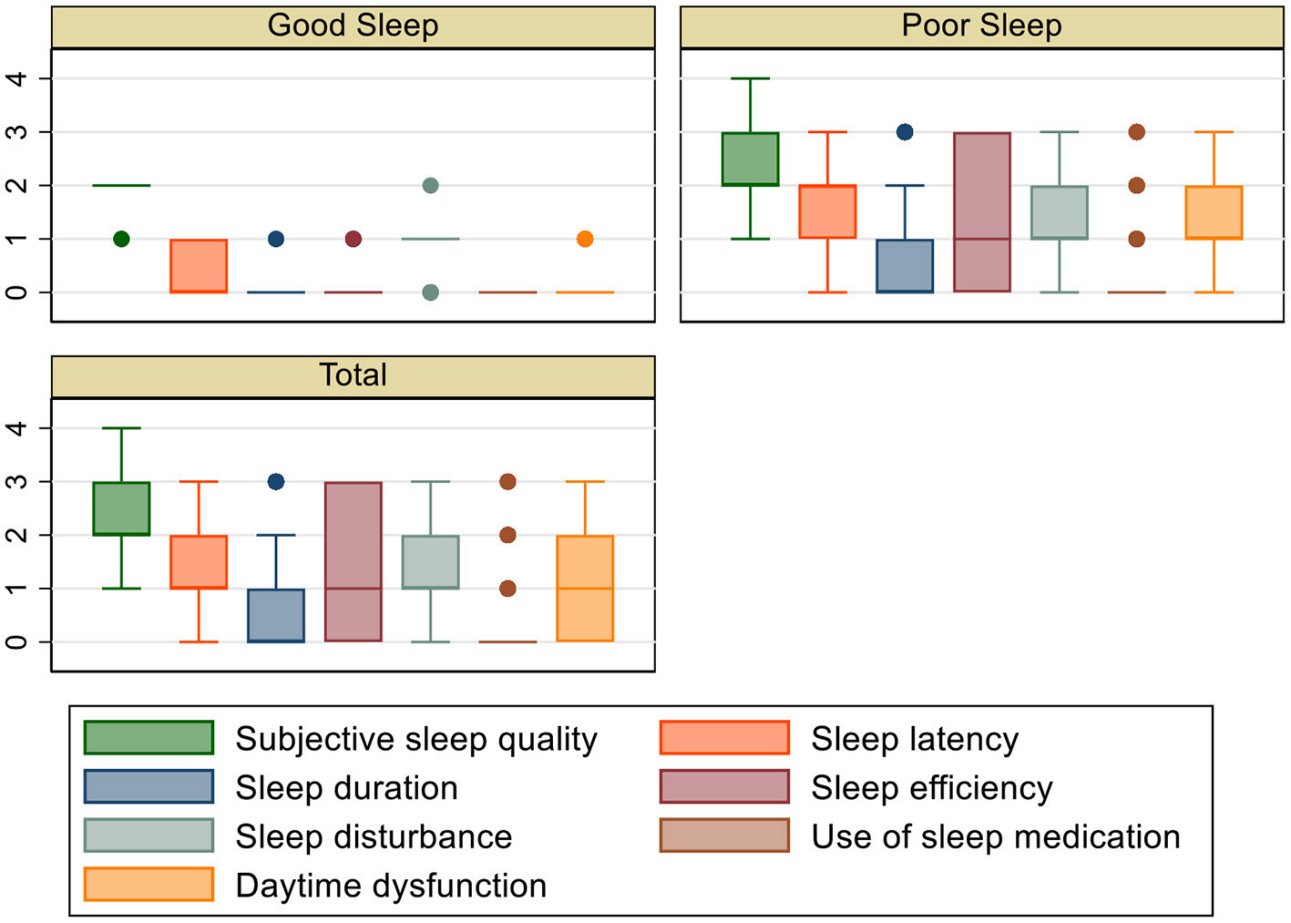
Box plot of PSQI components in participants with good and poor sleep.

The median PSQI score was 7 (6–10), and the median K10 score was 24 (18–31) (Table 2). The box plots of PSQI components in participants with good vs. poor sleep are presented in Figure 1. There was a significant difference in PSQI score between participants with poor vs. good sleep [8(6–10) vs. 4(3–4); *p* < 0.001] and between participants with distress vs. no distress [8(6–11) vs. 6(5–8); *p* < 0.001]. Participants with poor sleep had a higher K10 score compared to participants who had a good sleep [25(19–31) vs. 17(13–23); *p* < 0.001].

**Table 2:** PSQI score component and K-10 score.

**Variable**	**All participants (*n* = 836)**
Subjective sleep quality	2 (2–3)
Sleep latency	1 (1–2)
Sleep duration	0 (0–1)
Habitual sleep efficiency	1 (0–3)
Sleep disturbance	1 (1–2)
Use of sleeping medication	0 (0–0)
Day time dysfunction	1 (0–2)
PSQI score	7 (6–10)
K-10 score	24 (18–31)

*Continuous data were expressed as median and 25th−75th percentiles*.

### Factors Associated With Poor Sleep

Poor sleep was reported in 733 (87.7%) participants. Factors associated with poor sleep were recent changes in sleep habits due to special occasions such as Ramadan or vacations [(OR: 2.49(1.33–4.66); *p* = 0.004)], anxiety or fear because of coronavirus news on social media [2.13(1.10–4.11); *p* = 0.02], fear because there was no approved drug to treat COVID-19 [1.72(1.07–2.78); *p* = 0.03] and unawareness of the presence of chronic disease [9.15 (1.25–67.21); *p* = 0.03] (C-statistics: 0.65, Hosmer-Lemeshow *p* = 0.97). Recent changes in sleep habits, anxiety, fear because of coronavirus news on social media, chronic disease status, and hating social events were significantly associated with increased PSQI scores, while students had significantly lower PSQI scores (Table 3). Variables included in the multivariable logistic and negative binomial regressions are given in Supplementary Table 2.

**Table 3:** Factors associated with poor sleep and PSQI score.

	**Factors associated with poor sleep**		**Factors associated with PSQI score**	
	**OR (95% CI)**	***P***	**IRR (95% CI)**	***P***
Sleep habits affected by special occasions as Ramadan or vacations	2.49 (1.33–4.66)	0.004	1.19 (1.07–1.33)	0.001
Coronavirus news on social media increases my anxiety and fear (Highly agree)	2.13 (1.10–4.11)	0.02	1.05 (1.03–1.08)	<0.001
I have a chronic disease (I don't know)	9.15 (1.25–67.21)	0.03	1.05 (1.01–1.1)	0.02
I feel very afraid because there is no approved drug to treat COVID19 (Agree)	1.72 (1.07–2.78)	0.03	–	–
Hates social events and does not go there	–	–	1.17 (1.04–1.04)	0.01
Student	–	–	0.91 (0.84–0.98)	0.01

*CI, confidence interval; IRR, incidence rate ratio; OR, odds ratio*.

### Factors Associated With Distress

Distress was reported in 568 participants (67.9%). Female gender [OR: 1.54 (1.06–2.24); *p* = 0.02], living outside Riyadh (the capital) [OR: 1.74(1.23–2.48); *p* = 0.002], fear or anxiety because of coronavirus news on social media [OR: 1.64(1.02–2.65); *p* = 0.04], recent changes in sleep habits because of Ramadan or vacation [OR:1.97(1.15–3.39); *p* = 0.01], fear because there is no approved drug to treat coronavirus COVID-19 [OR:2.24(1.36–3.69); *p* = 0.001], monthly income of < SR 1000 [OR:2.07(1.22–3.5); *p* = 0.01], isolation [OR:2.08 (1.16–3.71); *p* = 0.01] and unaware of the presence of chronic disease [OR:2.53(1.19–5.4); *p* = 0.02] were associated with distress (C-statistics: 0.73, Hosmer-Lemeshow *p* = 0.17). Young age, students, and employees had lower K10 scores while getting bored or hating social events, fear of no available COVID-19 treatment, isolation, and feeling anxious or afraid of COVID-19 news on social media increased K10 score (Table 4). Variables included in the multivariable logistic and negative binomial regressions as given in Supplementary Table 3.

**Table 4:** Factors associated with distress and K-10 score.

	**Factors associated with distress**		**Factors associated with K-10 score**	
	**OR (95% CI)**	***P***	**IRR (95% CI)**	***P***
Age	0.97 (0.96–0.99)	0.003	0.99 (0.98–0.99)	<0.001
Female	1.54 (1.06–2.24)	0.02	–	–
Student	0.63 (0.4–0.99)	0.047	0.88 (0.82–0.95)	0.001
Employee	–	–	0.9 (0.84–0.96)	0.001
I feel very afraid because there is no approved drug to treat COVID19 (Highly agree)	2.24 (1.36–3.69)	0.001	1.05 (1.02–1.07)	<0.001
Coronavirus news on social media increases my anxiety and fear	0.65 (0.45–0.95)	0.03		
Disagree		–	–
Agree	1.64 (1.02–2.65)	–	1.13 (1.01–1.27)	0.04
Highly agree		0.04	1.26 (1.11–1.43)	<0.001
Sleep habits affected by special occasions as Ramadan or vacations	1.97 (1.15–3.39)	0.01	–	–
Living outside Riyadh	1.74 (1.23–2.48)	0.002	–	–
Monthly income <1000 SR	2.07 (1.22–3.5)	0.01	–	–
I have a chronic disease (I don't know)	2.53 (1.19–5.4)	0.02	–	–
Gets bored of social events and does not go there	–	–	1.12 (1.04–1.18)	0.002
Hates social events and does not go there	2.85 (1.27–6.4)	0.01	1.23 (1.12–1.35)	<0.001
Isolation	2.08 (1.16–3.71)	0.01	1.12 (1.04–1.21)	0.004

*CI, confidence interval; IRR, incidence rate ratio; OR, odds ratio*.

### Relationship Between Sleep and Distress

**Figure 2:**
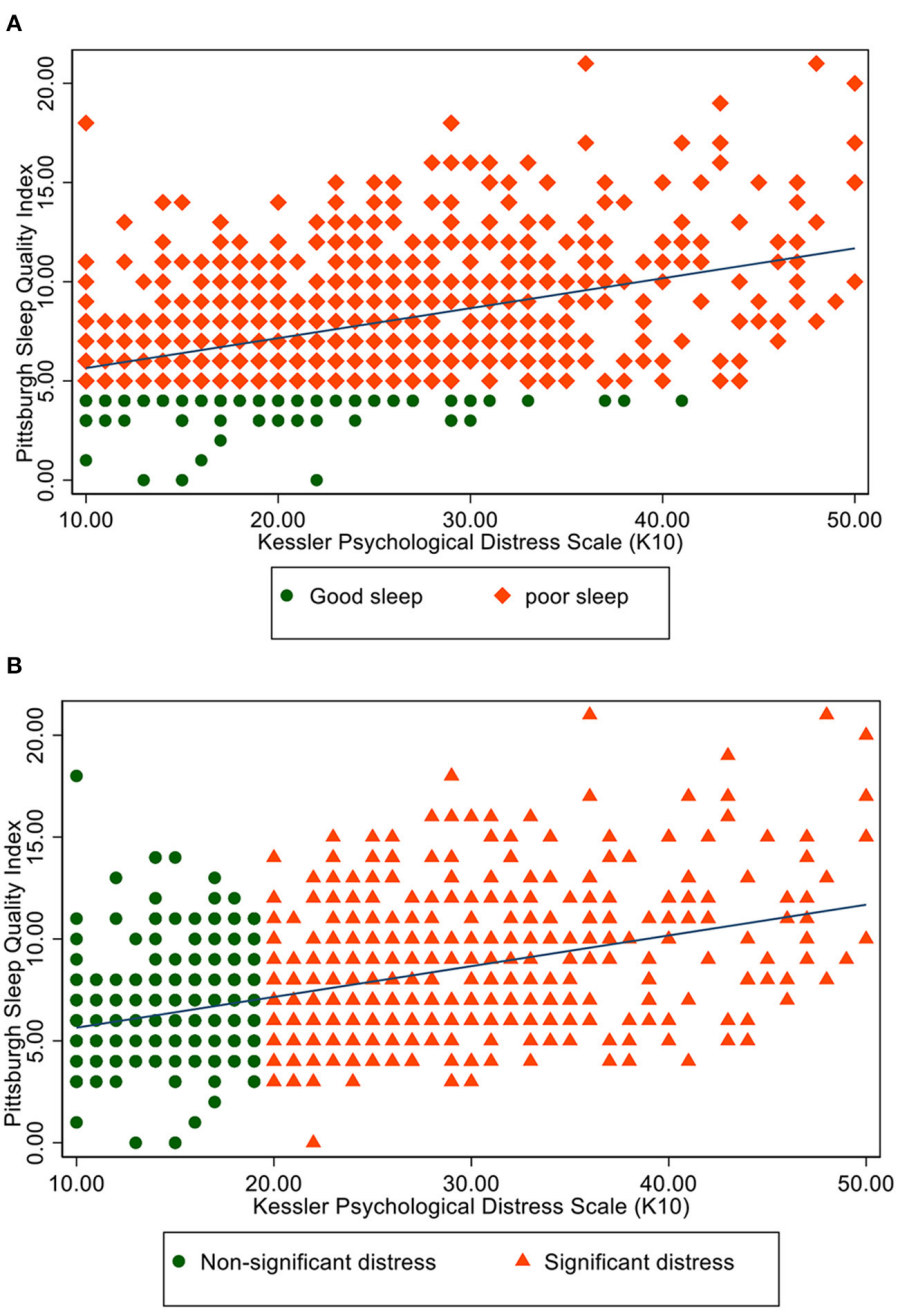
**(A)** Scatter plot of PSQI and K-10 scores in patients with poor vs. good sleep. **(B)** Scatter plot of PSQI and K-10 scores between participants with significant and non-significant distress.

**Figure 3:**
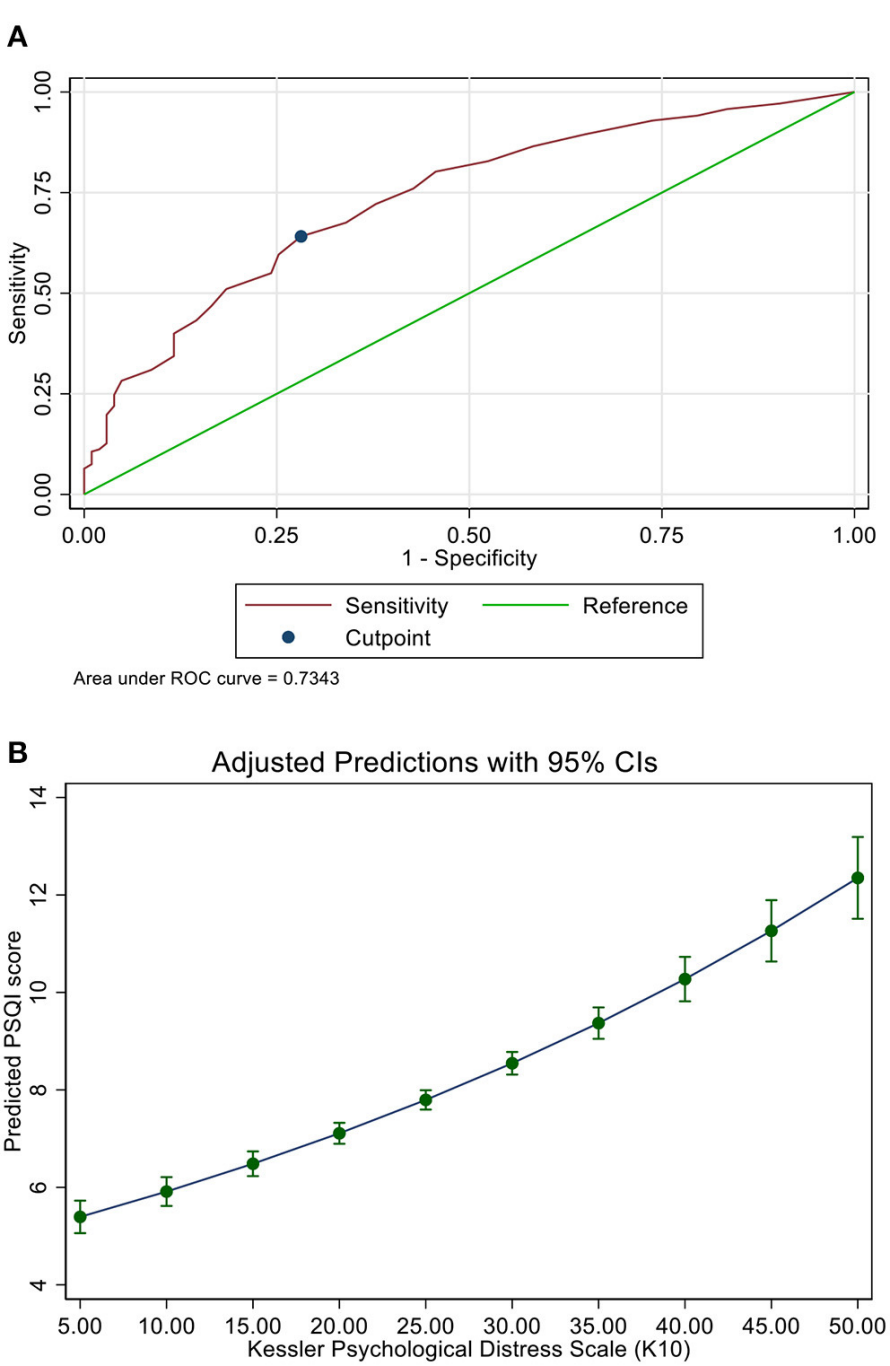
**(A)** The receiver operator curve (ROC) for the cut-off point of K-10 score predicting poor sleep. **(B)** The predicted PSQI score according to the K-10 score.

PSQI and K10 scores had a significant positive correlation (Spearman rho = 0.41; *p* < 0.001) (Figures 2A,B). A K10 score of 21 points predicted poor sleep with a sensitivity of 64%, specificity of 72%, area under the curve of 0.73 (Figure 3A). The predicted PSQI scores according to the measured K10 score are shown in Figure 3B.

**Figure 4:**
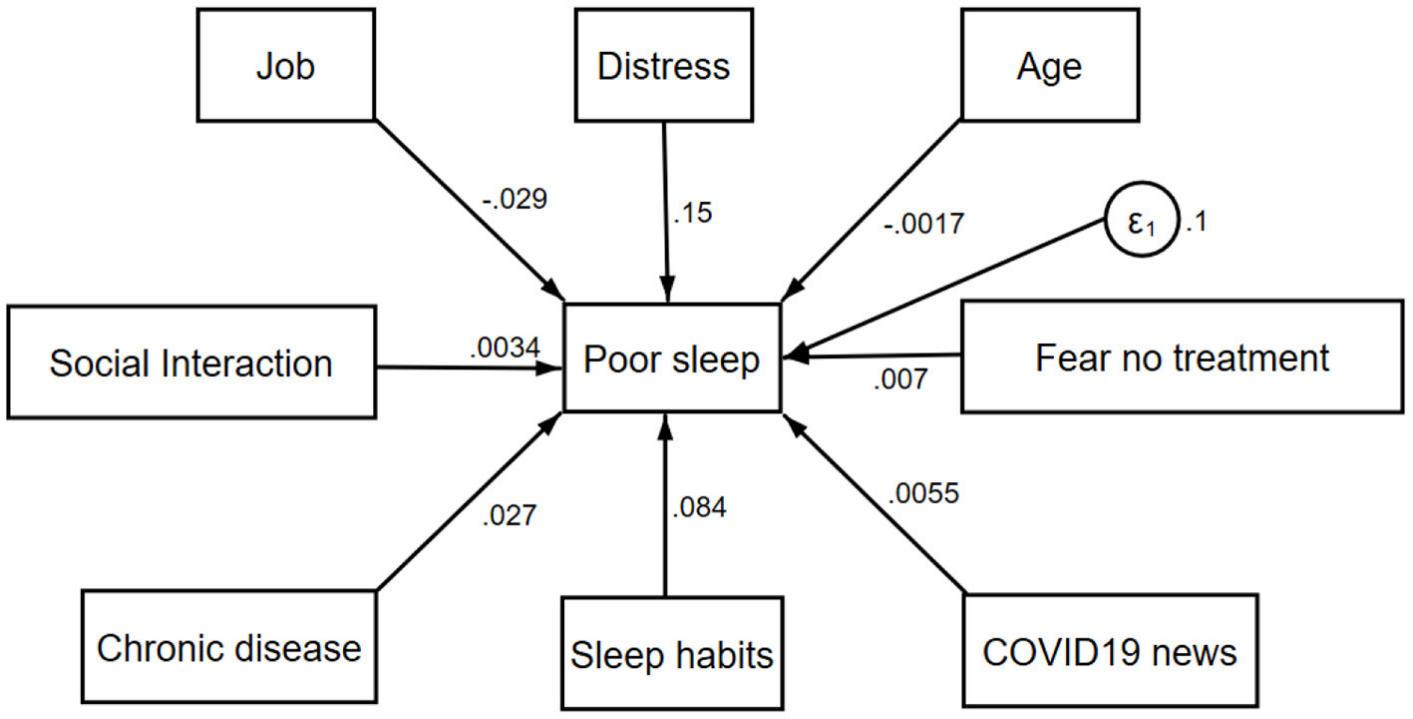
Relationship between poor sleep and factors associated with it (Numbers on arrows indicate the effect magnitude of each variable on sleep, ε is the calculated error).

Distress was included in a generalized structural equation model to evaluate its relationship with poor sleep in the presence of other variables. Distress was significantly associated with poor sleep [coefficient: 0.15(0.10–0.20); *p* < 0.001]. Additionally, recent changes in sleep habits increased poor sleep by 8.4% (*p* = 0.04), while students had lower chances of experiencing poor sleep (*p* = 0.01). All distress categories affected sleep significantly moderate [coefficient: 0.12 (0.05–0.17); *p* < 0.001], high [0.16 (0.1–0.23); *p* < 0.001], very high [0.18(0.12–0.24); *p* < 0.001. Other factors presented in Figure 4 were not significantly associated with poor sleep when distress was included in the model.

## Discussion

The current study found a high rate of sleep disturbances in the Saudi Arabia population during the COVID-19 lockdown, and that sleep disturbances increased the risk of mental health problems, particularly in front-line epidemic workers, people who were quarantined or isolated, young people. The findings emphasize the significance of interventions aimed at persons with sleep disorders in order to decrease mental health problems during a public health crisis. Vulnerable populations, in particular, should be continuously watched. The current findings can be used to establish mental health intervention policies during epidemic/pandemic situations.

An epidemic or pandemic such as the COVID-19 affects societies' physical and mental health (6, 7). During the COVID-19 outbreak, stress, anxiety, and depression increased, while sleep was similarly affected, as evidenced by various studies in different populations (4, 5, 8, 9, 11, 15, 16). Another study found that current or previous COVID-19 infection was associated with psychiatric disorders and loneliness (31). As a result, the objective of this study was to evaluate the factors that affected sleep quality and psychological distress in the Saudi population during the COVID-19 pandemic. Our study revealed that the prevalence of poor sleep was associated with recent changes in sleep habits, fear, and anxiety due to lack of approved drugs for treating the disease as well as an overflowing amount of COVID-19-related information on social media. Before COVID-19, medical residents in Saudi Arabia have a significant rate of poor sleep quality. The most mentioned sleep distractors were increased sleep latency and short sleep duration. Sleep deprivation was linked to on-call schedules and shift jobs. The 80-h weekly maximum for training programs should be adhered to, and wellness programs should be included in the curriculum (32). A similar trend was also reported in local studies that underlined deterioration in sleep quality and a high prevalence of sleep disorders during the spreading of the pandemic among physicians, quarantined individuals, and the public (33–36). Other studies from different countries have highlighted the increased prevalence of sleep problems. For example, Casagrande et al. (37) reported a 57.1% prevalence of poor sleep quality among the Italian population during the pandemic (37). Similarly, in a web-based cross-sectional survey of 7,236 Chinese individuals, Huang and Zhao (38) indicated that about 18% of the participants reported symptoms of poor sleep quality during the disease outbreak (38). An Italian cross-sectional study observed a significant increase in the PSQI score during COVID-19 lockdown (39).

The overwhelming COVID-19 social media news and information created fear and confusion among the public (40). Another study of 521 Bangladeshi individuals found that fear of the COVID-19 disease significantly impacted sleep quality, with significantly higher COVID-19 dread, perceived stress, and subjective sleep quality (41). In addition, poor sleep quality has a detrimental impact on life satisfaction, health, and social and emotional domains (42).

Our study found psychological distress among 67.9% of our sample, with a median K10 score of 24 (18–31). This finding is consistent with other studies that reported the increased prevalence of psychological distress during the pandemic (43–45). In some studies, the psychological distress related to pandemics has been associated with gender, whereby these trends among females appear to have remained constant or even become exasperated (46). Our study has also revealed a significant association of psychological distress with the female gender. These findings are comparable to the studies of Al-Hanawi et al. (45) and Alkhamees et al. (44), reporting similarly higher rates of distress among females during the pandemic (44, 45). The explanation for this might be that older individuals are better at managing their stress than younger ones because they better understand the epidemic. Another theory is that COVID-19 causes the most emotional anguish among younger individuals due to their high exposure to social media, which disseminates a significant quantity of information about the epidemic, some of which are important and some unsettling. Previous data from KSA supports this conclusion, demonstrating that internet addiction causes significant suffering among the young, particularly those at undergraduate college levels (45). In contrast, however, higher stress levels were linked to the disease in men than in women in some other studies, possibly pointing to ethnic or societal variations in such demographic-related analyses (47–49).

The COVID-19 epidemic expanded the use of electronic devices, particularly smartphones, as a method of reducing the negative consequences of social isolation and communicating with the rest of the world, all while preserving the necessity for social separation. As a result, the number of research documenting the negative impacts of excessive mobile device use on mental and physical health is continuously growing (50–54).

This study also revealed the sleep quality and psychological distress among healthcare workers during this outbreak of COVID-19. Previous national studies showed that healthcare workers are a vulnerable group susceptible to psychological distress (55, 56). Our results showed that almost 18% (*n* = 132) of the studied participants experienced poor sleep quality, and 16.9% reported psychological distress. Our findings concur with the recent data published from other countries. A recent analysis reported a 45.1% (95% CI: 37.2–53.1%) sleep disturbance and a higher total PSQI score of (9.83) in the Chinese healthcare workers during the pandemic (57).

Our study also observed a change in the prevalence of sleep quality and mental health among students. Findings indicate that 36.6% of students had poor sleep quality, while 40.5% experienced psychological distress. Comparable with our results, a recent study in Bangladesh reported that University students were mentally distressed and experiencing poor subjective sleep quality during the pandemic (58). Similar results were also revealed by Martinez-Lezaun et al. (59), who reported 70.7% of the University students showed worse sleep quality during the lockdown.

The recent COVID-19 pandemic has also triggered various economic crises that have resulted in psychological suffering among different groups of people in society. Accordingly, our study has shown significantly higher psychological distress among low-income categories. At the same time, a longitudinal study in the general Japanese population also reported severe psychological distress among those in the lower-income bracket compared to those in the higher category (60). A cross-national analysis from 62 countries found social isolation and loneliness adversely impact psychological wellbeing and its prediction of poor mental health of society (61) similar to our findings. A significant relationship was found in our study between sleep quality and psychological distress, as demonstrated by the significant positive correlation between PSQI scores and K10 scores. Similar findings were reported in different populations and risk groups (62, 63). Accordingly, it can be speculated that information linking sleep quality with psychological distress provides some important clues about the potential role of the former in predicting the onset of psychological problems and depressive disorders.

There were limitations to the current study that must be noted. First, the data and results were derived from a cross-sectional design; hence it is difficult to make causal inferences. Also, since this is a cross-sectional survey that was done during the early stages of the COVID-19 epidemic in Saudi Arabia, long-term effects are not known. The data particularly captures the mental health state at that moment. Second, using a web-based survey procedure to conduct a study such as ours within the period of social distancing limits the generalizability of the results. Most of our participants were relatively young, which could be due to the distribution of the survey through social media. This observation could lead to an underestimation of the psychological effect of the pandemic. Third, reporting bias is possible due to the self-reported nature of the survey. Fourth, since the median PSQI score is relatively low, this could be a particular kind of selection bias. Those who have voluntarily responded could be more interested in the topic since being sleep-disturbed. The survey did not include data related to contact with COVID-19 patients, which could be the source of stress. Several other factors could have affected sleep and were not included in the survey. Longitudinal follow-up studies are advised to investigate the dynamic dynamics of people's mental health state during the pandemic. Finally, no specialist sleep assessment instruments were utilized, which resulted in the omission of data such as the severity of sleep disorders, limiting our knowledge of the observed sleep abnormalities.

## Conclusion

Our survey results reveal a sizeable percentage of the Saudi population experienced poor sleep and psychological distress during the COVID-19 outbreak. Poor sleep was strongly associated with recent changes in sleep patterns, worry, or anxiety because of the lack of an authorized medication to treat coronavirus and the overabundance of information about COVID-19 on social media. In addition, distress was significantly correlated with female gender, low monthly income, and isolation, while sleep quality and psychological distress were interrelated.

## Data Availability Statement

Data are available upon reasonable requests to the corresponding author.

## Ethics Statement

The studies involving human participants were reviewed and approved by Institutional Review Board (IRB) of King Saud University Medical City (E-20-4869) and the IRB of the Ministry of Health (20-331E). The patients/participants provided their written informed consent to participate in this study.

## Author Contributions

MA, SA-A, HJ, and AB: conceptualization, investigation, and methodology. MA: formal analysis. MA, AA, KB, and NA: data curation. MA, SA-A, HJ, AB, NA, and FK: writing—original draft preparation, writing—review, and editing. All authors read and agreed to the published version of the manuscript.

## Conflict of Interest

The authors declare that the research was conducted in the absence of any commercial or financial relationships that could be construed as a potential conflict of interest. The reviewer FA declared a shared affiliation, with several of the authors MA, SA-A, AA, KB, FK, RA-k, and AB to the handling editor at the time of the review.

## Publisher's Note

All claims expressed in this article are solely those of the authors and do not necessarily represent those of their affiliated organizations, or those of the publisher, the editors and the reviewers. Any product that may be evaluated in this article, or claim that may be made by its manufacturer, is not guaranteed or endorsed by the publisher.
