# Supplementary material for: The Prevalence of Psychological Distress and Its Relationship to Sleep Quality in Saudi Arabia's General Population During the COVID-19 Pandemic
Source: Front Psychiatry. 2022 Feb 3;12:809040. doi: 10.3389/fpsyt.2021.809040 (PMC8851311; doi:10.3389/fpsyt.2021.809040)
Supplement: Supplementary file 1 [file Data_Sheet_1.docx]

**Appendix**

**Table 1.**Comparison of the socio-demographic data in participants with poor vs. good sleep and distress vs. no distress

| Variable | Poor sleep (n= 733) | Good sleep (n= 103) | P | Distress (n= 568) | No distress (n= 268) | P |
| --- | --- | --- | --- | --- | --- | --- |
| Age (Years) | 28 (22- 38) | 27 (22- 40) | 0.40 | 25 (21- 35) | 33 (23- 42) | <0.001 |
| Female | 553 (75.44%) | 71 (68.93%) | 0.15 | 443 (77.99%) | 181 (67.54%) | 0.001 |
| **Marital status**  Single  Married  Divorced/ widow/ separated | 405 (55.25%)  296 (40.38%)  32 (4.37%) | 59 (57.28%)  40 (38.83%)  4 (3.88%) | 0.95 | 341 (60.04%)  206 (36.27%)  21 (3.7%) | 123 (45.9%)  130 (48.51%)  15 (5.6%) | 0.001 |
| Do you work in the healthcare sector? (Yes) | 132 (18.01 %) | 26 (25.24%) | 0.08 | 96 (16.9%) | 62 (23.13%) | 0.03 |
| Have children | 282 (38.47%) | 40 (38.83%) | 0.94 | 196 (34.51%) | 126 (47.01%) | 0.001 |
| **How many members of your family live with you at home (including you)?**  One to two persons  Three to five persons  More than five persons | 86 (11.73%)  240 (32.74%)  407 (55.53%) | 17 (16.50%)  30 (29.13%)  56 (54.37%) | 0.36 | 73 (12.85%)  182 (32.04%)  313 (55.11%) | 30 (11.19%)  88 (32.84%)  150 (55.97%) | 0.79 |
| **Nationality**  *Saudi*  *Non-Saudi* | 676 (92.22%)  57 (7.78%) | 99 (96.12%)  4 (3.88%) | 0.22 | 524 (92.25%)  44 (7.75%) | 251 (93.66%)  17 (6.34%) | 0.47 |
| **Educational level**  *Middle school or lower, High school or Diploma*  *Bachelor's degree or higher* | 179 (24.42%)  554 (75.58%) | 17 (16.5%)  86 (83.5%) | 0.08 | 58 (21.64%)  210 (78.36%) | 58 (21.64%)  210 (78.36%) | 0.40 |
| **Job-status**  I do not work  Employee  Self-employed  Student | 144 (19.65%)  301 (41.06%)  20 (2.73%)  268 (36.56%) | 15 (14.56%)  40 (38.83%)  2 (1.94%)  46 (44.66%) | 0.41 | 116 (20.42%)  208 (36.62%)  14 (2.46%)  230 (40.49%) | 43 (16.04%)  133 (49.63%)  8 (2.99%)  84 (31.34%) | 0.003 |
| **Monthly income**  *I don't want to answer*  *< 1000 SR*  *1000-2999 SR*  *3000-5999 SR*  *6000-9999 SR*  *10000-30000 SR*  *> 30000* | 266 (36.29%)  122 (16.64%)  72 (9.82%)  44 (6%)  51 (6.96%)  152 (20.74%)  26 (3.55%) | 49 (47.57%)  14 (13.59%)  9 (8.74%)  0  6 (5.83%)  19 (18.45%)  6 (5.83%) | 0.03 | 212 (37.32%)  114 (20.07%)  57 (10.04%)  28 (4.93%)  40 (7.04%)  104 (18.31%)  13 (2.29%) | 103 (38.43%)  22 (8.21%)  24 (8.96%)  16 (5.97%)  17 (6.34%)  67 (25%)  19 (7.09%) | <0.001 |
| Region (Riyadh) | 471 (64.26%) | 65 (63.11%) | 0.82 | 341 (60.04%) | 195 (72.76%) | <0.001 |
| **Social interaction**  *Loves and waits for social events*  *Gets bored of social events and does not go there*  *Hates social events and does not go there*  *Neutral* | 254 (34.65%)  141 (19.24%)  47 (6.41%)  291 (39.7%) | 47 (45.63%)  21 (20.39%)  7 (6.8%)  28 (27.18%) | 0.80 | 189 (33.27%)  112 (19.72%)  46 (8.1%)  221 (38.91%) | 112 (41.79%)  50 (18.66%)  8 (2.99%)  98 (36.57%) | 0.01 |
| **How often do you go out weekly before the coronavirus pandemic outside working hours?**  None  Once a week  Two to three times a week  Four times or more | 66 (9%)  150 (20.46%)  287 (39.15%)  230 (31.38%) | 9 (8.74%)  28 (27.18%)  38 (36.89%)  28 (27.18%) | 0.47 | 52 (9.15%)  120 (21.13%)  222 (39.08%)  174 (30.63%) | 23 (8.58%)  58 (21.64%)  103 (38.43%)  84 (31.34%) | 0.99 |
| **I have good information about coronavirus and its ways of spreading**  *Highly agree*  *Agree*  *Neutral*  *Disagree*  *Highly disagree* | 640 (87.31%)  18 (2.46%)  61 (8.32%)  11 (1.5%)  3 (0.41%) | 93 (90.29%)  2 (1.94%)  7 (6.80%)  1 (.97%)  0 | 0.98 | 497 (87.5%)  10 (1.76%)  48 (8.45%)  11 (1.94%)  2 (0.35%) | 236 (88.06%)  10 (1.76%)  20 (7.46%)  1 (.37%)  1 (0.37%) | 0.16 |
| **I feel very afraid because there is no approved drug to treat COVID19**  *Highly agree*  *Agree*  *Neutral*  *Disagree*  *Highly disagree* | 133 (18.14%)  270 (36.83%)  174 (23.74%)  127 (17.33%)  29 (3.96%) | 19 (18.45%)  26 (25.24%)  26 (25.24%)  21 (20.39%)  11 (10.68%) | 0.01 | 126 (22.18%)  207 (36.44%)  125 (22.01%)  86 (15.14%)  24 (4.23%) | 26 (9.7%)  89 (33.21%)  75 (27.99%)  62 (23.13%)  16 (5.97%) | <0.001 |
| **Coronavirus news on social media increases my anxiety and fear**  *Highly agree*  *Agree*  *Neutral*  *Disagree*  *Highly disagree* | 158 (21.56%)  237 (32.33%)  152 (20.74%)  149 (20.33%)  37 (5.05%) | 11 (10.68%)  34 (33.01%)  24 (23.3%)  24 (23.3%)  10 (9.71%) | 0.05 | 139 (24.47%)  196 (34.51%)  118 (20.77%)  95 (16.73%)  20 (3.52%) | 30 (11.19%)  75 (27.99%)  58 (21.64%)  78 (29.1%)  27 (10.07%) | <0.001 |
| Isolated | 73 (9.96%) | 11 (10.68%) | 0.82 | 66 (11.62%) | 18 (6.72%) | 0.03 |
| **Do you have COVID-19?**  Yes  No  In the past | 21 (2.86%)  654 (89.22%)  58 (7.91%) | 3 (2.91%)  91 (88.35%)  9 (8.74%) | 0.89 | 14 (2.46%)  500 (88.03%)  54 (9.51%) | 10 (3.73%)  245 (91.42%)  13 (4.85%) | 0.046 |
| **Curfew hours during the past month**  *Partial curfew 6 am – 3 pm*  *Partial curfew 6 am – 8 pm penalties for not wearing a face mask*  *No curfew, penalties for not wearing a face mask, refuse to be checked for temperature* | 369 (50.34%)  361 (49.25%)  3 (0.41%) | 55 (53.4%)  48 (46.6%)  0 | 0.73 | 269 (47.36%)  297 (52.29%)  2 (0.35%) | 155 (57.84%)  112 (41.79%)  1 (0.37%) | 0.02 |
| Pregnancy | 16 (2.18%) | 3 (2.91%) | 0.72 | 10 (1.76%) | 9 (3.36%) | 0.15 |
| Are your sleep habits affected by special occasions as Ramadan or vacations? (Yes) | 681 (92.91%) | 87 (84.47%) | 0.003 | 533 (93.84%) | 235 (87.69%) | 0.002 |
| **Do you suffer from a chronic disease?**  *No*  *Yes*  *I don't know* | 586 (79.95%)  88 (12.01%)  59 (8.05%) | 85 (82.52%)  17 (16.5%)  1 (0.97%) | 0.01 | 460 (80.99%)  57 (10.04%)  51 (8.98%) | 211 (78.73%)  48 (17.91%)  9 (3.36%) | <0.001 |

Continuous data were expressed as median (25^th^- 75^th^ percentiles) and categorical data as numbers and percentages.

**Appendix**

**Table 2.**Variables included in the multivariable logistic and negative binomial regression analyses for factors affecting poor sleep and PSQI score

| Variable | P-value |
| --- | --- |
| ***Logistic regression analysis:*** |  |
| Ccoronavirus news on social media increases my anxiety and fear | 0.01 |
| Sleep habits affected by special occasions as Ramadan or vacations | 0.004 |
| I feel very afraid because there is no approved drug to treat COVID19 | 0.02 |
| Monthly income | <0.001 |
| Educational level | 0.08 |
| Job-status | 0.10 |
| Age | 0.15 |
| I have a chronic disease | 0.10 |
| ***Negative binomial regression analysis:*** |  |
| Ccoronavirus news on social media increases my anxiety and fear | <0.001 |
| Sleep habits affected by special occasions as Ramadan or vacations | <0.001 |
| I feel very afraid because there is no approved drug to treat COVID19 | <0.001 |
| Social interaction | 0.049 |
| Isolation | 0.1 |
| I have a chronic disease | 0.02 |
| Age | 0.08 |
| Do you have children? | 0.12 |
| Nationality | 0.16 |
| Job | 0.02 |
| Region where you live | 0.09 |

**Appendix**

**Table 3.**Variables included in the multivariable logistic and negative binomial regression analyses for factors affecting distress and K-10 score

| Variable | P-value |
| --- | --- |
| ***Logistic regression analysis*** |  |
| Social interaction | 0.003 |
| I feel very afraid because there is no approved drug to treat coronavirus COVID19 | <0.001 |
| Coronavirus news on social media increases my anxiety and fear | <0.001 |
| Isolation | 0.03 |
| Do you have COVID-19? | 0.06 |
| Curfew hours during the past month | 0.006 |
| Pregnant | 0.15 |
| Your sleep habits affected by special occasions as Ramadan or vacations | 0.003 |
| Suffer from a chronic disease | 0.01 |
| Gender | 0.001 |
| Age | <0.001 |
| Marital status | <0.001 |
| Health sector worker | 0.03 |
| Job-status | 0.12 |
| Monthly income | 0.002 |
| The region where you live | <0.001 |
| Having children | 0.001 |
| ***Negative binomial regression*** |  |
| Social interaction | <0.001 |
| I feel very afraid because there is no approved drug to treat coronavirus COVID19 | <0.001 |
| Coronavirus news on social media increases my anxiety and fear | <0.001 |
| Isolation | 0.01 |
| Do you have COVID-19? | 0.03 |
| Curfew hours during the past month | <0.001 |
| Your sleep habits affected by special occasions as Ramadan or vacations | 0.01 |
| Age | <0.001 |
| Marital status | <0.001 |
| Healthcare worker | 0.14 |
| Job | 0.03 |
| Monthly income | <0.001 |
| The region where you live | 0.001 |
| Having children | <0.001 |
| Education | 0.08 |
