# Supplementary material for: The Prevalence of Psychological Distress and Its Relationship to Sleep Quality in Saudi Arabia's General Population During the COVID-19 Pandemic
Source: Front Psychiatry. 2022 Feb 3;12:809040. doi: 10.3389/fpsyt.2021.809040 (PMC8851311; doi:10.3389/fpsyt.2021.809040)
Supplement: Supplementary file 2 [file Data_Sheet_2.docx]

**Supplementary Material:** **Survey questionnaire**

1. Sociodemographic data

1.How often do you go out weekly before the coronavirus pandemic outside working hours?

- None
- Once per week
- Two to three times a week
- Four times or more

2. I consider myself a person who?

- Has many friends
- Has one or two friends
- Has no friends

3. I consider myself a person who?

- loves and waits for social events
- Neutral
- gets bored of social occasions
- Gets bored of social events and does not go there
- hates social events and does not go there

4. I have good information about coronavirus and its ways of spreading?

- Highly agree
- Agree
- Neutral
- Disagree
- Highly disagree

5. I feel very afraid because there is no approved drug to treat coronavirus COVID-19 ?

- Highly agree
- Agree
- Neutral
- Disagree
- Highly disagree

6.Coronavirus news on social media increases my anxiety and fear?

- Highly agree
- Agree
- Neutral
- Disagree

7. Currently I am ?

- Not subjected to the country duo to the curfew lift
- Subjected to the quarantine restrictions (at my home) because of the curfew law
- Subjected to quarantine restrictions (at my home) because of coronavirus suspicion
- Subjected to quarantine restrictions (at my home) because of coronavirus infection
- subjected to isolation restrictions (at my home) because of arrival from abroad
- Subjected to isolation restrictions (at the quarantine facility as hospital or hotel) because of coronavirus suspicion
- Subjected to isolation restrictions (at the quarantine facility as hospital or hotel) because of coronavirus infection
- ‏Subjected to isolation restrictions (at the quarantine facility as hospital or hotel) because of arrival from abroad

8. Isolation Vs None ?

- Isolation
- None

9. Do you have Coronavirus COVID-19 ?

- Yes
- No
- I don’t know

10. Since when you are in quarantine or isolation facility (days) ?

__________________________________

11. Curfew hours during the past month?

__________________________________

12. Curfew hours during the past month?

- Partial curfew 6 am – 3 pm
- Partial curfew 6 am – 8 pm. Penalties for not wearing face mask
- No curfew, Penalties for not wearing face mask, refuse to be checked for temperature when entering public or private sector, fail to adhere to social distancing rules

13. I am currently in the quarantine or isolation facility due to?

- Country curfew
- Coronavirus COVID-19 infection
- Coronavirus COVID 19 suspicion
- I am not at at quarantine or isolation

14. Are you pregnant?

- Yes
- No
- Not applicable

15. Are your sleep habits affected by special occasions as Ramadan or vacations?

- Yes
- No

16. Do you suffer from a chronic disease?

- Yes
- No
- I don’t know

17. Do you take medicines for one of these diseases؟

- Sleep problems
- Psychiatric illnesses
- None

1. Personal Information

1.Gender:

- Male
- Female

2. Age

____________________

3. Marital Status:

- Single
- Married
- Divorced/widowed /separated

4. Do you work in a health sector

- Yes
- No

5. Number of children

- 0
- 1
- 2
- 3
- 4 or more

6. How many members of your family live with you at home (including you)

- One to two persons
- Three to five persons
- More than five persons

7.Nationality:

- Saudi
- Non-Saudi

8. Job Status:

- I don’t work
- Employee
- Self employed
- Student

9.Education Level

- Middle school or lower
- High school
- Diploma
- Bachelor's degree
- Master's degree or higher

10. The country where I live

- Saudi Arabia
- Other

11. Work venue during the coronavirus pandemic

- I go to my workplace
- Home
- I don’t work
